# Insignificant Effect of Isolated Hypothyroxinemia on Pregnancy Outcomes During the First and Second Trimester of Pregnancy

**DOI:** 10.3389/fendo.2020.528146

**Published:** 2020-10-16

**Authors:** Liangmiao Chen, Hong Yang, Enling Ye, Zhenzhen Lin, Mengmeng Peng, Hai Lin, Lechu Yu, Zhuhua Cai, Xuemian Lu

**Affiliations:** ^1^Department of Endocrinology, Shandong Provincial Hospital, Cheeloo College of Medicine, Shandong University, Jinan, China; ^2^Department of Endocrinology, Third Affiliated Hospital of Wenzhou Medical University, Ruian, China; ^3^Department of Obstetrics and Gynecology, Center for Reproductive Medicine, Qilu Hospital, Cheeloo College of Medicine, Shandong University, Jinan, China

**Keywords:** isolated hypothyroxinemia, first trimester, second trimester, pregnancy outcome, maternal outcome, perinatal complication

## Abstract

**Objective:** Adverse maternal outcomes and perinatal complications are associated with overt and subclinical maternal hypothyroidism. It is not clear whether these complications also occur in women with isolated hypothyroxinemia during pregnancy. The aim of this study was to evaluate the effects of isolated hypothyroxinemia on maternal and perinatal outcomes during pregnancy.

**Methods:** This study included data from 2,864 pregnant women in the first trimester (67 women with isolated hypothyroxinemia, 784 euthyroid women) and the second trimester (70 women with isolated hypothyroxinemia, 1,943 euthyroid women) of pregnancy. Maternal serum samples were collected in the first and second trimesters to examine thyroid hormone concentration. Hypothyroxinemia was defined as a normal maternal thyroid-stimulating hormone concentration with a low maternal free thyroxine concentration and negative thyroid autoantibodies. The following maternal outcomes were recorded: gestational hypertension, gestational diabetes mellitus, placenta previa, placental abruption, prelabor rupture of membranes, and premature delivery. Perinatal outcomes, including fetal growth restriction, fetal distress, low birth weight, intrauterine fetal death, and malformation. The incidence of adverse pregnancy outcomes and perinatal complications was compared between women in the first trimester and second trimester with isolated hypothyroxinemia.

**Results:** There were no significant differences in the incidence rates of adverse maternal outcomes and perinatal complications between patients in the first and second trimesters with isolated hypothyroxinemia.

**Conclusion:** The results of this study indicate that isolated hypothyroidism does not increase the incidence of adverse maternal outcomes and perinatal complications.

## Introduction

Thyroid hormone is required for growth, development, energy metabolism, and temperature regulation in humans (1). In the first trimester of pregnancy, thyroid hormone is supplied from the mother to the fetus *via* the placenta. In the second trimester, although the fetus begins to synthesize thyroid hormone, it still obtains thyroid hormone mainly from the mother. Therefore, hypothyroidism during the first trimester of pregnancy affects the growth of the fetus. Hypothyroidism during pregnancy includes clinical hypothyroidism, hypothyroxinemia, and subclinical hypothyroidism. Hypothyroxinemia is defined as a normal maternal thyroid-stimulating hormone (TSH) concentration in conjunction with a low maternal free thyroxine (FT4) concentration. Studies have reported different cutoff levels for FT4, which can be at the lower fifth or 10th percentile of a given population (2). Isolated hypothyroxinemia refers to hypothyroxinemia with negative thyroid autoantibodies. Hypothyroidism during pregnancy can induce adverse outcomes (3). A number of studies have demonstrated the effects of subclinical hypothyroidism on pregnancy outcomes and the fetus (4–6). While there are many studies reporting the effect of elevated TSH concentrations on adverse pregnancy outcome, few studies have investigated the effect of isolated hypothyroxinemia on adverse pregnancy outcomes (7). A Brazilian study discovered that hypothyroxinemia in the first trimester of gestation is associated with poor pregnancy and neonatal outcomes (8). Reports indicate that hypothyroxinemia during pregnancy is associated with adverse pregnancy outcomes, but the adverse pregnancy outcomes observed in different studies are not consistent (9–11). Few studies with a large sample size have been conducted on the Chinese population. Gong et al. (12) observed that hypothyroxinemia during pregnancy was related to adverse pregnancy outcomes, but the effects of hypothyroxinemia during the first and second trimesters on pregnancy outcome were not consistent. The 2017 American Thyroid Association (ATA) guidelines for the diagnosis and management of thyroid disease during pregnancy and postpartum highlighted the lack of evidence regarding the association between hypothyroxinemia during pregnancy and fetal development and stated that the benefits of interventions to treat hypothyroxinemia have not been demonstrated (7). Therefore, treatment of hypothyroxinemia during pregnancy is not recommended by ATA guidelines, although the European Thyroid Association guidelines recommend the treatment of hypothyroidism in early pregnancy based on the importance of thyroid hormone in fetal nervous system development during early pregnancy. This study was conducted in China and assessed a large cohort of patients to investigate the impact of isolated hypothyroxinemia during the first and second trimesters on pregnancy outcomes.

## Subjects and Methods

### Subjects

A total of 3,911 women in the first and second trimesters of pregnancy with a single fetus who were undergoing antenatal examination between August 2010 and June 2014 in the Obstetrical Department of the Third Hospital Affiliated to Wenzhou Medical University, China, were screened. After excluding women with hyperthyroidism, hypothyroidism, hyperthyroxinemia, and positive thyroid autoantibodies, a total of 3,050 women with euthyroid function and negative thyroid autoantibodies as well as hypothyroxinemia were included in the study. All the participants were local residents or had resided in Wenzhou city for at least 5 years. The study exclusion criteria were as follows: (1) pregnant women who were being treated or had a past history of treatment with thyroid hormone or antithyroid drugs; (2) pregnant women with a history of other autoimmune diseases; (3) pregnant women with congenital heart disease or cardiac insufficiency; (4) pregnant women with increased serum transaminase and serum creatinine levels; (5) tumor or tumor history; or (6) pregnant women in a state of stress, such as after injury or infection.

## Methods

This study was approved by the Ethics Committee of the Third Hospital Affiliated to Wenzhou Medical University. The protocol was approved by the Ethics Committee of the Third Hospital Affiliated to Wenzhou Medical University (Ref. No. LZM2020008). During antenatal examination, all women were asked routine questions regarding age, medical history, and history of childbirth. Fasting venous blood was collected in the morning, and the serum was isolated and cryopreserved at −80°C for further laboratory analysis. Detection of TSH, FT4, thyroglobulin antibody (TGAb), and thyroid peroxidase antibody (TPOAb) in serum samples was performed using an automated chemiluminescence immune analyzer (DX2800, Beckman Corp., Germany). The coefficient of variation (CV) for within-run assays and for between-run assays were <10%. Before enrollment, thyroid function, TPOAb, and TGAb levels were assessed in all participants as part of the antenatal examination. Blood samples were taken at 6–13 gestational weeks for the first trimester and at 14–27 gestational weeks for the second trimester. If the assessment results were not consistent with the clinical results, a review was conducted within 1 week for confirmation. After enrollment, all participants attended the hospital every 4 weeks, and blood pressure, oral glucose tolerance test, and obstetric ultrasound scan results were recorded. Gestational age (recorded in completed weeks) at delivery and birth weight were also recorded.

### Diagnosis of Isolated Hypothyroxinemia

In a previous study, reference ranges for thyroid function during pregnancy were determined for pregnant women in Wenzhou District as follows (both TSH and FT4 ranged between 2.5 and 97.5%): first trimester, TSH 0.09–3.47 mIU/L and FT4 6.00–12.25 ng/L; second trimester, TSH 0.20–3.81 mIU/L and FT4 4.30–9.74 ng/L; and late pregnancy, TSH 0.67–4.99 mIU/L and FT4 4.56–8.50 ng/L (13). In this study, the diagnostic criteria for normal thyroid function during pregnancy were as follows: TSH, 2.5–97.5% and FT4, 10–90%. Accordingly, in the first trimester, FT4 was 6.89–10.39 ng/L, and in the second trimester, FT4 was 4.94–8.14 ng/L. The diagnostic criteria for hypothyroxinemia were as follows: TSH, 2.5–97.5% and FT4 10% lower than the reference range above. The criteria for negative thyroid autoantibody were as follows: TPOAb <50 IU/ml and TGAb <115 IU/ml.

### Definitions of Adverse Pregnancy Outcomes

The following definitions of adverse pregnancy outcomes, based on medical guidelines and the reported literature, were used in this study: (1) premature birth: gestational week at delivery ranged from 28 to <37 weeks; (2) placenta previa: the placenta was attached to the lower uterine segment and was located below the presenting part of the fetus; (3) placental abruption: the placenta was partially or completely separated from the uterine wall before childbirth; (4) fetal growth restriction: an estimated fetal weight below the 10th percentile for gestational age; (5) intrauterine fetal death: fetus died *in utero* after 20 weeks of pregnancy; (6) fetal distress: fetal heart rate of <120 or >160 beats/min, presence of meconium, signs of abnormal fetal movement, fetal scalp pH of 7.2, and fetal abnormalities or complications resulting from a lack of oxygen supply; (7) premature rupture of the membranes: the amniotic sac and chorion membranes ruptured before labor; (8) low birth weight: birth weight <2,500 g; (9) pregnancy-induced hypertension: no history of hypertension before pregnancy; systolic blood pressure ≥140 mmHg and/or diastolic blood pressure ≥90 mmHg after 20 weeks of pregnancy, including eclampsia and preeclampsia (14); (10) gestational diabetes mellitus: plasma glucose concentration ≥92 mg/dl after fasting, ≥180 mg/dl at 1 h after a 75-g oral glucose tolerance test (OGTT), and/or ≥153 mg/dl at 2 h after a 75-g OGTT, regardless of gestational age (15); (11) fetal malformations, including deformity of the eyes, ears, face, nervous system, circulatory system, urogenital system, skeletal system, and other organs.

### Statistical Analysis

Data processing and analysis were performed using SPSS 17.0 software. Data were analyzed using the *t*-test, the *X*^2^ test was used for ratio comparison, and the risk factors were analyzed by binary logistic regression analysis. A *P* < 0.05 was considered statistically significant.

## Results

A total of 3,911 women in the first and second trimesters of pregnancy were screened. Of these, 3,050 met the enrollment criteria and were included in this study. There were 897 women in the first trimester. Of these, 20 were lost to follow-up because of emigration, 11 declined follow-up, and 15 had an abortion. Finally, out of 851 women in the first trimester of pregnancy, 67 had isolated hypothyroxinemia (observation group) and 784 had normal euthyroid function (control group). There were 2,153 women in the second trimester. Of these, 87 were lost to follow-up because of emigration, 42 declined follow-up, and 11 had an abortion. Finally, out of 2,013 women in the second trimester of pregnancy, 70 had isolated hypothyroxinemia (observation group) and 1,943 had euthyroid function (control group). In this study, the incidence of isolated hypothyroxinemia during the first and second trimesters of pregnancy was 4.8% (137/2,864). The baseline data and adverse pregnancy outcomes in the observation and control groups during the first and second trimesters were analyzed.

### The First Trimester of Pregnancy

#### Baseline Data Comparison

There were no significant differences in TSH level, age, gestational age, and cesarean section ratio between the observation group and the control group (*P* > 0.05). FT4 levels in the observation group were significantly lower than those in the control group (*P* < 0.001; Table 1).

**Table 1:** Baseline data of euthyroid women and those with isolated hypothyroxinemia in the first trimester of pregnancy.

**Variables**	**Observation group (*n* = 67)**	**Control group (*n* = 784)**	***P*-value**
Age of pregnant women	27.2 ± 3.3	26.5 ± 3.5	0.124
Gestational week at child birth	39.0 ± 1.5	39.0 ± 1.6	0.956
Cesarean section ratio	46.4% (37/67)	55.2% (364/784)	0.202
TSH	1.41 ± 0.70	1.25 ± 0.74	0.086
FT4	6.22 ± 0.59	8.62 ± 0.88	<0.001

*TSH, thyroid-stimulating hormone; FT4, serum free thyroxine*.

#### Comparison of Incidence of Adverse Pregnancy Outcomes

The adverse maternal outcomes and perinatal complications included placenta previa, placental abruption, premature birth, fetal distress, intrauterine fetal death, fetal malformation, low birth weight, gestational diabetes mellitus, pregnancy-induced hypertension, fetal growth restriction, and premature rupture of the membranes. There was no significant difference in the incidence of adverse pregnancy outcomes between the observation group and the control group (*P* > 0.05; Table 2).

**Table 2:** Incidence of adverse pregnancy outcomes of euthyroid women and those with isolated hypothyroxinemia in the first trimester of pregnancy.

**Outcome**	**Control group (*n* = 784)**	**Observation group (*n* = 67)**	***P*-value**
Gestational diabetes mellitus	54 (6.9%)	4 (6.0%)	0.973
Pregnancy-induced hypertension	7 (0.9%)	1 (1.5%)	0.483
Placenta previa	0	0	-
Placental abruption	0	1 (1.5%)	0.079
Fetal growth restriction	6 (0.8%)	1 (1.5%)	0.438
Fetal distress	22 (2.8%)	1 (1.5%)	0.807
Intrauterine fetal death	4 (0.5%)	0	-
Fetal malformation	3 (0.4%)	1 (1.5%)	0.280
Premature rupture of membranes	229 (29.2%)	18 (27.3%)	0.088
Premature birth	19 (2.4%)	2 (3.0%)	0.678
Low birth weight infants	8 (1.0%)	2 (3.0%)	0.183

#### Analysis of Risk Factors of Adverse Pregnancy Outcomes

Taking placental abruption, premature birth, fetal distress, intrauterine fetal death, fetal malformation, low birth weight, gestational diabetes mellitus, pregnancy-induced hypertension, fetal growth restriction, and premature rupture of the membranes as the dependent variables and maternal age, gestational week, TSH, and FT4 as the independent variables, binary logistic regression analysis showed no correlation between those adverse pregnancy outcomes and FT4 (Table 3).

**Table 3:** Correlation analysis between adverse pregnancy outcome and FT4 in the first trimester of pregnancy.

**Outcome**	**OR**	**95% CI**	***P*-value**
Gestational diabetes mellitus	0.919	0.717–1.178	0.505
Pregnancy-induced hypertension	0.867	0.468–1.604	0.649
Placental abruption	0.125	0.010–1.617	0.111
Fetal growth restriction	0.883	0.461–1.692	0.708
Fetal distress	1.336	0.872–2.046	0.183
Intrauterine fetal death	1.106	0.420–2.910	0.838
Fetal malformation	0.566	0.281–1.142	0.112
Premature rupture of membranes	1.029	0.894–1.183	0.694
Premature birth	1.049	0.694–1.586	0.820
Low birth weight infants	0.652	0.378–1.127	0.126

### The Second Trimester of Pregnancy

#### Baseline Data Comparison

There were no significant differences in TSH level, age, gestational age, and cesarean section ratio between the observation group and the control group (*P* > 0.05). FT4 levels in the observation group were significantly lower than those in the control group (*P* < 0.001; Table 4).

**Table 4:** Baseline data of euthyroid women and those with isolated hypothyroxinemia in the second trimester of pregnancy.

**Variables**	**Observation group (*n* = 70)**	**Control group (*n* = 1,943)**
Age of pregnant women	28.0 ± 4.0	27.2 ± 3.7
Gestational week at child birth	39.0 ± 1.1	39.0 ± 1.4
Cesarean section ratio	61.4% (43/70)	49.9% (969/1943)
TSH	1.55 ± 0.67	1.47 ± 0.68
FT4	4.43 ± 0.64	6.71 ± 0.80

*TSH, thyroid-stimulating hormone; FT4, serum free thyroxine*.

#### Comparison of Incidence of Adverse Pregnancy Outcomes

The adverse maternal outcomes and perinatal complications included placenta previa, placental abruption, fetal distress, intrauterine fetal death, fetal malformation, premature birth, low birth weight, gestational diabetes mellitus, pregnancy-induced hypertension, fetal growth restriction, and premature rupture of membranes. There was no significant difference in the incidence of adverse outcomes between the observation group and the control group (*P* > 0.05; Table 5).

**Table 5:** Incidence of adverse pregnancy outcomes of euthyroid women and those with isolated hypothyroxinemia in the second trimester of pregnancy.

**Outcome**	**Control group (*n* = 1,943)**	**Observation group (*n* = 70)**	***P*-value**
Gestational diabetes mellitus	107 (5.5%)	7 (10.0%)	0.182
Pregnancy-induced hypertension	21 (1.1%)	1 (1.4%)	0.543
Placenta previa	3 (0.2%)	1 (1.4%)	0.132
Placental abruption	2 (0.1%)	0	-
Fetal growth restriction	22 (1.1%)	1 (1.4%)	0.559
Fetal distress	64 (3.3%)	5 (7.1%)	0.160
Intrauterine fetal death	3 (0.2%)	0	-
Fetal malformation	8 (0.4%)	1 (1.4%)	0.273
Premature rupture of membranes	17 (0.9%)	2 (2.9%)	0.140
Premature birth	49 (2.5%)	2 (2.9%)	0.420
Low birth weight infants	32 (1.6%)	1 (1.4%)	0.235

#### Analysis of Risk Factors of Adverse Pregnancy Outcomes

Taking placental abruption, premature birth, fetal distress, intrauterine fetal death, fetal malformation, low birth weight, gestational diabetes mellitus, pregnancy-induced hypertension, fetal growth restriction, and premature rupture of the membranes as the dependent variables and maternal age, gestational week, TSH, and FT4 as the independent variables, binary logistic regression analysis showed no correlation between those adverse pregnancy outcomes and FT4 (Table 6).

**Table 6:** Correlation analysis between adverse pregnancy outcome and FT4 in the second trimester of pregnancy.

**Outcome**	**OR**	**95% CI**	***P*-value**
Gestational diabetes mellitus	1.049	0.847–1.299	0.660
Pregnancy-induced hypertension	0.660	0.435–1.003	0.051
Placenta previa	0.914	0.295–2.828	0.876
Placental abruption	1.026	0.992–1.061	0.133
Fetal growth restriction	0.959	0.601–1.530	0.861
Fetal distress	1.059	0.807–1.390	0.678
Intrauterine fetal death	1.008	0.979–1.013	0.579
Fetal malformation	0.878	0.425–1.815	0.726
Premature rupture of membranes	0.659	0.419–1.036	0.071
Premature birth	1.238	0.889–1.724	0.207
Low birth weight infants	1.022	0.660–1.583	0.923

The diagnostic criteria for normal thyroid function during pregnancy could be as follows: TSH 2.5–97.5% and FT4 5–95%, and the diagnostic criteria for hypothyroxinemia were as follows: TSH 2.5–97.5% and FT4 5% lower than the reference range above. However, there was still no correlation between adverse pregnancy outcomes and FT4 (Tables 7–12).

**Table 7:** Baseline data of euthyroid women and those with isolated hypothyroxinemia in the first trimester of pregnancy.

**Variables**	**Observation group (*n* = 40)**	**Control group (*n* = 882)**	***P*-value**
Age of pregnant women	27.6 ± 3.6	26.5 ± 3.5	0.067
Gestational week at child birth	39.0 ± 1.5	39.0 ± 2.0	0.897
Cesarean section ratio	55% (22/40)	51% (450/882)	0.622
TSH	1.37 ± 0.72	1.24 ± 0.74	0.281
FT4	5.90 ± 0.57	8.73 ± 1.07	<0.001

*TSH, thyroid-stimulating hormone; FT4, serum free thyroxine*.

**Table 8:** Incidence of adverse pregnancy outcomes of euthyroid women and those with isolated hypothyroxinemia in the first trimester of pregnancy.

**Outcome**	**Control group (*n* = 882)**	**Observation group (*n* = 40)**	***P*-value**
Gestational diabetes mellitus	58 (6.6%)	2 (5.6%)	1
Pregnancy-induced hypertension	9 (1.0%)	0	-
Placenta previa	0	0	-
Placental abruption	1 (0%)	1 (2.5%)	0.085
Fetal growth restriction	8 (0.9%)	0	-
Fetal distress	25 (2.8%)	1 (2.5%)	1
Intrauterine fetal death	4 (0.5%)	0	-
Fetal malformation	3 (0.5%)	2 (5.0%)	0.017
Premature rupture of membranes	246 (27.9%)	2 (5.0%)	0.001
Premature birth	20 (2.3%)	1 (2.5%)	0.610
Low birth weight infants	10 (1.2%)	1 (2.5%)	0.415

**Table 9:** Correlation analysis between adverse pregnancy outcome and FT4 in the first trimester of pregnancy.

**Outcome**	**OR**	**95% CI**	***P*-value**
Gestational diabetes mellitus	0.859	0.691–1.069	0.173
Pregnancy-induced hypertension	0.797	0.455–1.396	0.427
Placental abruption	0.805	0.259–2.501	0.708
Fetal growth restriction	0.804	0.446–1.450	0.469
Fetal distress	1.318	0.932–1.862	0.118
Intrauterine fetal death	0.933	0.412–2.116	0.868
Fetal malformation	0.582	0.315–1.076	0.084
Premature rupture of membranes	0.891	0.788–1.008	0.067
Premature birth	0.920	0.640–1.324	0.655
Low birth weight infants	0.630	0.373–1.061	0.082

**Table 10:** Baseline data of euthyroid women and those with isolated hypothyroxinemia in the second trimester of pregnancy.

**Variables**	**Observation group (*n* = 37)**	**Control group (*n* = 2,187)**
Age of pregnant women	27.9 ± 3.9	27.2 ± 3.7
Gestational week at child birth	39.3 ± 1.0	39.0 ± 1.4
Cesarean section ratio	62.2% (23/70)	50.1% (1096/2187)
TSH	1.53 ± 0.76	1.46 ± 0.68
FT4	4.09 ± 0.73	6.84 ± 0.94

*TSH, thyroid-stimulating hormone; FT4, serum free thyroxine*.

**Table 11:** Incidence of adverse pregnancy outcomes of euthyroid women and those with isolated hypothyroxinemia in the second trimester of pregnancy.

**Outcome**	**Control group (*n* = 2,187)**	**Observation group (*n* = 37)**	***P*-value**
Gestational diabetes mellitus	119 (5.4%)	1 (2.7%)	0.465
Pregnancy-induced hypertension	22 (1.0%)	0	-
Placenta previa	4 (0.2%)	1 (2.7%)	0.081
Placental abruption	2 (0.1%)	0	-
Fetal growth restriction	23 (1.1%)	0	-
Fetal distress	73 (3.3%)	4 (10.8%)	0.037
Intrauterine fetal death	3 (0.1%)	0	-
Fetal malformation	9 (0.4%)	0	-
Premature rupture of membranes	26 (1.2%)	2 (5.4%)	0.078
Premature birth	55 (2.5%)	2 (5.4%)	0.245
Low birth weight infants	36 (1.6%)	0	-

**Table 12:** Correlation analysis between adverse pregnancy outcome and FT4 in the second trimester of pregnancy.

**Outcome**	**OR**	**95% CI**	***P*-value**
Gestational diabetes mellitus	0.958	0.797–1.152	0.652
Pregnancy-induced hypertension	0.743	0.497–1.110	0.147
Placenta previa	1.121	0.445–2.823	0.809
Placental abruption	2.975	0.473–18.724	0.245
Fetal growth restriction	0.796	0.531–1.194	0.271
Fetal distress	1.061	0.842	1.061
Intrauterine fetal death	0.550	0.112–2.698	0.550
Fetal malformation	0.747	0.396–1.408	0.367
Premature rupture of membranes	1.153	0.782–1.700	0.471
Premature birth	1.189	0.902–1.567	0.221
Low birth weight infants	0.909	0.624–1.323	0.617

## Discussion

The 2017 ATA guidelines state that physiological changes in pregnant women affect thyroid hormone levels (3). Moreover, variation in thyroid hormone levels has been found according to race, geographical region, and detection methods and reagents (7). Therefore, it is important to determine an appropriate unit or region-specific thyroid function reference range during pregnancy. The present study was based on a regional specific thyroid function reference range for the first and second trimesters of pregnancy established in a previous study, which examined the diagnosis of thyroid function during pregnancy (13). The prevalence of hypothyroxinemia calculated at different FT4 cutoff values varies in different populations, ranging between 2 and 9.5% with an FT4 cutoff <5% (12). In our study, the prevalence of hypothyroxinemia was 4.8% (FT4 <10% below the reference range). This is lower than results reported by Hamm et al. (16) (10.1%). However, the authors did not exclude hypothyroxinemia in their study by screening for positive thyroid autoantibodies.

Fetal thyroid function is barely established during the first and second trimesters of pregnancy so thyroid hormone required for fetal development derives almost completely from the mother. Therefore, maternal thyroid hormone levels during the first and second trimesters have a significant influence on brain and fetal development. Hypothyroxinemia is a state of mild hypothyroidism with an unclear etiology. Studies have reported that the most common cause of hypothyroxinemia is iodine deficiency or autoimmune thyroiditis (17), although this has not been clearly established. The adverse effects of hypothyroxinemia on pregnancy outcomes and the perinatal period remain controversial. This study found that the incidence of adverse pregnancy outcomes was not increased in pregnant women with isolated hypothyroxinemia compared with pregnant women with euthyroid function. Casey et al. (18) analyzed serum TSH, T4, and TPOAb levels of 17,298 pregnant women during the first half of pregnancy and found that there was no significant difference in the incidence of placental abruption, premature birth, neonatal asphyxia, and pregnancy-induced hypertension in the hypothyroxinemia group compared with the normal control group; furthermore, TSH level did not correlate with low FT4 levels, indicating that hypothyroxinemia in pregnancy had no obvious adverse effect on pregnancy outcomes. Hamm et al. (16) examined the association between hypothyroxinemia in the first trimester and pregnancy outcomes using indicators such as premature birth, neonatal Apgar score, small for gestational age, and *z*-score value of birth weight. Hypothyroxinemia was found to have no adverse effect on pregnancy outcomes or fetal development.

However, other studies have reported contradictory findings. Gong et al. (12) observed that hypothyroxinemia was not related to adverse pregnancy outcomes in the first trimester of pregnancy, although the risk of pregnancy-induced hypertension and macrosomia was increased in mothers with hypothyroidism during the second trimester. The precise correlation between hypothyroxinemia and pregnancy outcomes is difficult to establish, as hypothyroxinemia during different stages of pregnancy results in different outcomes. Fetal development in the first trimester of pregnancy is more dependent on maternal FT4 levels than in the second trimester. This appears to contradict the finding that the low FT4 levels in the first trimester do not increase the adverse pregnancy outcome rate, while low FT4 levels in the second trimester are significantly associated with the adverse pregnancy outcome. Su et al. (19) found that hypothyroxinemia in the Chinese population was associated with fetal distress, premature birth, spontaneous abortion, and motor-system malformations. Therefore, the authors postulated that hypothyroxinemia may affect early embryonic development; however, the number of subjects included in the study was small and only 43 women were identified with hypothyroxinemia. Positive thyroid antibodies may have adverse effects on pregnancy outcomes and fetal development (20, 21). A study in Henan, China, found that women with isolated hypothyroxinemia had significantly increased odds of macrosomia [adjusted odds ratio (aOR) 2.22, 95% CI 1.13–4.85]. However, a limitation of the study was that blood samples were obtained at different stages of gestation (22).

This study included a larger sample size and was involved a Chinese population. Stratification was performed according to first and second trimesters of pregnancy, and the interference effect of thyroid autoantibodies was excluded. There were no significant differences in the incidence rates of adverse maternal outcomes and perinatal complications between the first and second trimesters, which contradicts previously published data. However, this study has some limitations. First, it was a single-center study and the generalizibility of results must be approached with caution. Second, the number of cases of hypothyroxinemia during the first and second trimesters of gestation was relatively small and no offspring follow-up was performed.

There is a paucity of evidence regarding the effect of gestational hypothyroxinemia on pregnancy outcomes, and findings reported in authoritative guidelines, including ATA guidelines, and the medical literature are inconsistent; it is not clear from the reported data whether intervention in pregnant women with hypothyroxinemia is recommended (7). Further evidence-based studies are required. The present study indicated that isolated hypothyroxinemia in the first and second trimesters of pregnancy does not have an adverse effect on pregnancy outcomes, indicating that screening for hypothyroxinemia and intervention are not required during pregnancy.

## Conclusion

Isolated hypothyroxinemia in the first and second trimesters of pregnancy does not adversely affect pregnancy outcomes, indicating that screening for hypothyroxinemia and intervention are not required during pregnancy. Further prospective, large sample size, and multicenter studies are required.

## Data Availability Statement

The datasets generated for this study are available on request to the corresponding author.

## Ethics Statement

The studies involving human participants were reviewed and approved by the Ethics Committee of Third Hospital Affiliated to Wenzhou Medical University, China. Written informed consent was obtained from all the participants of the study.

## Author Contributions

XL was responsible for the conception, design, and completion of the entire study. LC analyzed the data and wrote the manuscript. HY analyzed the data. EY, ZL, MP, HL, LY, and ZC collected the data. All authors contributed to the article and approved the submitted version.

## Conflict of Interest

The authors declare that the research was conducted in the absence of any commercial or financial relationships that could be construed as a potential conflict of interest.

## References

[1] Laurberg P Andersen SL Pedersen IB Andersen S Carle A. Screening for overt thyroid disease in early pregnancy may be preferable to searching for small aberrations in thyroid function tests. Clin Endocrinol. (2013) 79:297–304. 10.1111/cen.12232 23627986

[2] Furnica RM Lazarus JH Gruson D Daumerie C. Update on a new controversy in endocrinology: isolated maternal Hypothyroxinemia. J Endocrinol Invest. (2015) 38:117–23. 10.1007/s40618-014-0203-5 25370910

[3] Stagnaro-Green A. Overt hyperthyroidism and hypothyroidism during pregnancy. Clin Obstet Gynecol. (2011) 54:478–87. 10.1097/GRF.0b013e3182272f32 21857178

[4] Barišić T Mandić V Vasilj A Tiric D. Higher levels of thyrotropin in pregnancy and adverse pregnancy outcomes. J Matern Fetal Neonatal Med. (2019) 32:2883–8. 10.1080/14767058.2018.1451509 29540085

[5] Chen LM Du WJ Dai J Zhang Q Si GX Yang H. Effects of subclinical hypothyroidism on maternal and perinatal outcomes during pregnancy: a single-center cohort study of a Chinese population. PLoS ONE. (2014) 9:e109364. 10.1371/journal.pone.0109364 25353960 PMC4212915

[6] Consortium on Thyroid Pregnancy—Study Group on Preterm Birth Korevaar TIM Derakhshan A Taylor PN Meima M Chen L. Association of thyroid function test abnormalities and thyroid autoimmunity with preterm birth: a systematic review and meta-analysis. JAMA. (2019) 322:632–41. 10.1001/jama.2019.10931 31429897 PMC6704759

[7] Alexander EK Pearce EN Brent GA Brown RS Chen H Dosiou C. Guidelines of the american thyroid association for the diagnosis and management of thyroid disease during pregnancy and the postpartum. Thyroid. (2017) 27:315–89. 10.1089/thy.2016.0457 28056690

[8] Rosario PW Oliveira LFF Calsolari MR. Maternal hypothyroxinemia in the first trimester of gestation and association with obstetric and neonatal outcomes and iron deficiency: a prospective Brazilian study. Arch Endocrinol Metab. (2018) 62:332–36. 10.20945/2359-3997000000043 29791654 PMC10118786

[9] León G Murcia M Rebagliato M Álvarez-Pedrerol M Castilla AM Basterrechea M. Maternal thyroid dysfunction during gestation, preterm delivery, and birthweight. The infancia y medio ambiente cohort, Spain. Paediatr Perinat Epidemiol. (2015) 29:113–22. 10.1111/ppe.12172 25565408

[10] Haddow JE Craig WY Neveux LM Haddow HR Palomaki GE Lambert-Messerlian G. Implications of High Free Thyroxine (FT4) concentrations in euthyroid pregnancies: the FaSTER trial. J Clin Endocrinol Metab. (2014) 99:2038–44. 10.1210/jc.2014-1053 24606107 PMC4037729

[11] Korevaar TI Schalekamp-Timmermans S de Rijke YB Visser WE Visser W de Muinck Keizer-Schrama SM. Hypothyroxinemia and TPO-antibody positivity are risk factors for premature delivery: the generation R study. J Clin Endocrinol Metab. (2013) 98:4382–90. 10.1210/jc.2013-2855 24037884

[12] Gong X Liu A Li Y Sun H Li Y Li C. The impact of isolated maternal hypothyroxinemia during the first and second trimester of gestation on pregnancy outcomes: an intervention and prospective cohort study in China. J Endocrinol Invest. (2019) 42:599–607. 10.1007/s40618-018-0960-7 30334197 PMC6476837

[13] Lu XM Chen LM Yang H Du WJ Lin H Ye E Trimester specific reference data and variation of thyroid hormones for normal pregnancy. J Med Res. (2012) 41:70–3.

[14] Mancia G Fagard R Narkiewicz K Redon J Zanchetti A Böhm M (2013). ESH/ESC guidelines for the management of arterial hypertension: the task force for the management of arterial hypertension of the European Society of Hypertension (ESH) and of the European Society of Cardiology (ESC). Eur Heart J. (2013) 34:2159–219. 10.1093/eurheartj/eht151 23771844

[15] World Health Organization Diagnostic criteria and classification of hyperglycaemia first detected in pregnancy: a World Health Organization Guideline. Diabetes Res Clin Pract. (2014) 103:341–63. 10.1016/j.diabres.2013.10.012 24847517

[16] Hamm MP Cherry NM Martin JW Bamforth F Burstyn I. The impact of isolated maternal hypothyroxinemia on perinatal morbidity. J Obstet Gynaecol Can. (2009) 31:1015–21. 10.1016/S1701-2163(16)34345-6 20175339

[17] López-Muñoz E Mateos-Sánchez L Mejía-Terrazas GE Bedwell-Cordero SE. Hypothyroidism and isolated hypothyroxinemia in pregnancy, from physiology to the clinic. Taiwan J Obstet Gynecol. (2019) 58:757–63. 10.1016/j.tjog.2019.09.005 31759523

[18] Casey BM Dashe JS Spong CY McIntire DD Leveno KJ Cunningham GF. Perinatal significance of isolated maternal hypothyroxinemia identified in the first half of pregnancy. Obstet Gynecol. (2007) 109:1129–35. 10.1097/01.AOG.0000262054.03531.24 17470594

[19] Su PY Huang K Hao JH Xu YQ Yan SQ Li T. Maternal thyroid function in the first twenty weeks of pregnancy and subsequent fetal and infant development: a prospective population-based cohort study in China. J Clin Endocrinol Metab. (2011) 96:3234–41. 10.1210/jc.2011-0274 21832110

[20] Thangaratinam S Tan A Knox E Kilby MD Franklyn J Coomarasamy A. Association between thyroid autoantibodies and miscarriage and preterm birth: meta-analysis of evidence. BMJ. (2011) 342:d2616. 10.1136/bmj.d2616 21558126 PMC3089879

[21] Chen LM Zhang Q Si GX Chen QS Ye EL Yu LC. Associations between thyroid autoantibody status and abnormal pregnancy outcomes in euthyroid women. Endocrine. (2015) 48:924–8. 10.1007/s12020-014-0420-x 25209893

[22] Yang J Liu Y Liu HJ Zheng HM Li XF Zhu L. Associations of maternal iodine status and thyroid function with adverse pregnancy outcomes in Henan Province of China. J Trace Elem Med Biol. (2018) 47:104–10. 10.1016/j.jtemb.2018.01.013 29544795

